# Research Progress of Anti-PD-1/PD-L1 Immunotherapy Related Mechanisms and Predictive Biomarkers in NSCLC

**DOI:** 10.3389/fonc.2022.769124

**Published:** 2022-02-09

**Authors:** Fenglong Bie, He Tian, Nan Sun, Ruochuan Zang, Moyan Zhang, Peng Song, Lei Liu, Yue Peng, Guangyu Bai, Bolun Zhou, Shugeng Gao

**Affiliations:** ^1^ Department of Thoracic Surgery, National Cancer Center/National Clinical Research Center for Cancer/Cancer Hospital, Chinese Academy of Medical Sciences and Peking Union Medical College, Beijing, China; ^2^ State Key Laboratory of Molecular Oncology, National Cancer Center/National Clinical Research Center for Cancer/Cancer Hospital, Chinese Academy of Medical Sciences and Peking Union Medical College, Beijing, China

**Keywords:** non-small cell lung cancer (NSCLC), programmed cell death-1 (PD-1)/programmed death-ligand 1 (PD-L1), immunotherapy, predictive biomarkers, drug resistance

## Abstract

Programmed cell death-1 (PD-1)/programmed death-ligand 1 (PD-L1) is an important pair of immune checkpoints (IC), which play an essential role in the immune escaping process of tumors. Anti-PD-1/PD-L1 immunotherapy can block the suppression effect of the immune system produced by tumor cells through the PD-1/PD-L1 axis and restore the pernicious effect of the immune system on tumor cells. The specific mechanism of anti-PD-1/PD-L1 immunotherapy is closely related to PI3K (phosphatidylinositol 3-kinase)/AKT (AKT serine/threonine kinase 1), JNK (c-Jun N-terminal kinase), NF-kB (nuclear factor-kappa B subunit 1), and other complex signaling pathways. Patients receiving anti-PD-1/PD-L1 immunotherapy are prone to drug resistance. The mechanisms of drug resistance mainly include weakening recognition of tumor antigens by immune cells, inhibiting activation of immune cells, and promoting the production of suppressive immune cells and molecules. Anti-PD-1/PD-L1 immunotherapy plays a vital role in non-small cell lung cancer (NSCLC). It is essential to find better efficacy prediction-related biomarkers and screen patients suitable for immunotherapy. At present, common biomarkers related to predicting immune efficacy mainly include PD-L1 expression level in tumors, tumor mutation burden (TMB), microsatellite instability (MSI)/mismatch repair (MMR), mutations of driver gene, etc. However, the screening efficacy of each indicator is not ideal, and the combined application of multiple indicators is currently used. This article comprehensively reviews anti-PD-1/PD-L1 immunotherapy-related mechanisms, drug resistance-related mechanisms, and therapeutic efficacy-related predictive biomarkers.

## Introduction

Lung cancer is the tumor with the second morbidity rate and the first mortality rate globally (1, 2). Non-small cell lung cancer (NSCLC) accounts for 85% of lung cancer (3). At present, the main treatments for NSCLC are surgery, radiotherapy, chemotherapy, and targeted therapy (3–5). With the rise of immunotherapy in recent years, immunotherapy has also become one of the main treatments for NSCLC (6). Programmed cell death-1 (PD-1, also called *CD279*)/programmed death-ligand 1 (PD-L1, also known as *CD274*) is a pair of critical immune checkpoints (IC) and tumor cells can inhibit the killing effect of the immune system by activating the PD-1/PD-L1 axis (7). The anti-PD-1/PD-L1 immunotherapy can block the PD-1/PD-L1 axis and restore the lethal effect of the immune system on tumor cells (8). After binding of PD-1 to PD-L1, it can antagonize T cell (antigen) receptor (TCR) recognition by phosphorylating the Src homology 2 domain tyrosine phosphatases (SHP-2) site and block T cells from functioning (9). The activity of T cells is inhibited, leading to immune escaping of tumor cells through phosphatidylinositol 3-kinase (PI3K)/AKT serine/threonine kinase 1 (AKT)/mammalian target of rapamycin (mTOR) and other signaling pathways (10).

Patients receiving immunotherapy often develop drug resistance. Current studies have shown that drug resistance occurs mainly through weakening recognition of tumor antigens by immune cells, inhibiting activation of immune cells, and promoting the production of suppressive immune cells and molecules (11, 12). The current common prediction markers of immunotherapy effect include PD-L1 expression level of tumor tissue, tumor mutation burden (TMB), microsatellite instability (MSI)/mismatch repair (MMR), related driver gene mutations, etc. But the predictive effect of each marker is not ideal, and a combination of multiple indicators is usually used (13–15).

Given current research on anti-PD-1/PD-L1 immunotherapy, we have found some problems. Some patients have higher PD-L1 expression, but the therapeutic effect is not ideal. On the contrary, some patients have lower or even negative PD-L1 expression, but the therapeutic effect is well. Biomarkers such as TMB, Tumor Proportion Score (TPS), MSI/MMR, driver gene mutations are not perfect for evaluating the efficacy of immunotherapy. At present, there is no ideal predictive biomarker for the therapeutic effect of immunotherapy, and a combination of multiple indicators is usually used to improve the predictive efficiency. According to reports, the current effective rate of immunotherapy in lung cancer is not more than 30% (16), and the overall effective rate is just about 20% (17). Therefore, it is essential to find suitable biomarkers for immune efficacy prediction and screen out appropriate patients for immunotherapy. Based on the above questions, we want to explore anti-PD-1/PD-L1 immunotherapy efficacy and drug resistance related mechanisms in NSCLC. At the same time, we hope to find more reliable biomarkers for predicting immunotherapy efficacy. We have made a comprehensive review of these issues.

## Mechanisms Related to the Efficacy of anti-PD-1/PD-L1 Immunotherapy

### Structure and Expression of PD-1 and PD-L1

The IC proteins currently discovered include PD-1, PD-L1, cytotoxic T lymphocyte-associated molecule-4 (CTLA-4), lymphocyte-activation gene 3 (LAG3), T cell immunoglobulin, mucin domain-containing protein 3 (TIM3), etc. But the most researched ICs are PD-1 and PD-L1 (18, 19). PD-1 is a transmembrane protein encoded by human programmed cell death protein 1 (*PDCD1*) (20, 21), which is mainly expressed on the surface of activated T cells, B cells, and natural killer (NK) cells (22). PD-1 can exert an immunosuppressive effect by combining PD-L1/programmed cell death 1 ligand 2 (PD-L2, *CD273*) (23). Both PD-L1 and PD-L2 belong to the B7 protein family (24). PD-L1 is mainly expressed on the surface of tumor cells, immune cells, epithelial cells, and endothelial cells. In contrast, PD-L2 is primarily expressed on the surface of dendritic cells and macrophages (25). PD-L1 is more widely expressed on normal cells and tumor cells, and its role is much more significant than that of PD-L2, so most of the current studies focus on PD-L1 (26).

Many studies focus on the protein expression level of PD-L1 in tumor cells. Most of these studies have shown that the level of PD-L1 expression is closely related to the efficacy of anti-PD-1/PD-L1 immunotherapy (27, 28). The commonly used parameter in NSCLC to reflect the expression level of PD-L1 is the TPS score, which is the percentage of positive tumor cells. TPS= (PD-L1 staining positive tumor cells)/(total live tumor cells) *100% (29). PD-L1 expression levels can be divided into two types, innate and adaptive immune expression. The expression mode of innate immunity is regulated by genes and has a certain fixity (19). The expression mode of adaptive immunity is affected by immune and inflammatory factors, such as interleukin, interferon, tumor necrosis factor, etc. This expression mode will change with the dynamic changes of the tumor immune microenvironment (19).

### Mechanisms of PD-1/PD-L1 Inhibitors

The tumor immunoediting processes are divided into three steps: immune elimination, immune balance, and immune escape (12, 30). PD-1/PD-L1 are essential immune checkpoints, which can act as a brake on the immune system and play a crucial role in the immune escape process of tumors (31). A combination of PD-1 and PD-L1 can generate immunosuppressive signals and inhibit the follow-up effects of the immune system (32). PD-L1 molecules are highly expressed on the surface of tumor cells. PD-L1 binds to PD-1 molecules on the surface of immune cells (mainly T lymphocytes), which can induce immune cell exhaustion and inhibit the direct killing effect of immune cells on tumors. It can induce immune cells (primarily T helper cells) to secrete immunosuppressive factors to inhibit further the immune system’s killing effect on tumor cells (33).

**Figure 1:**
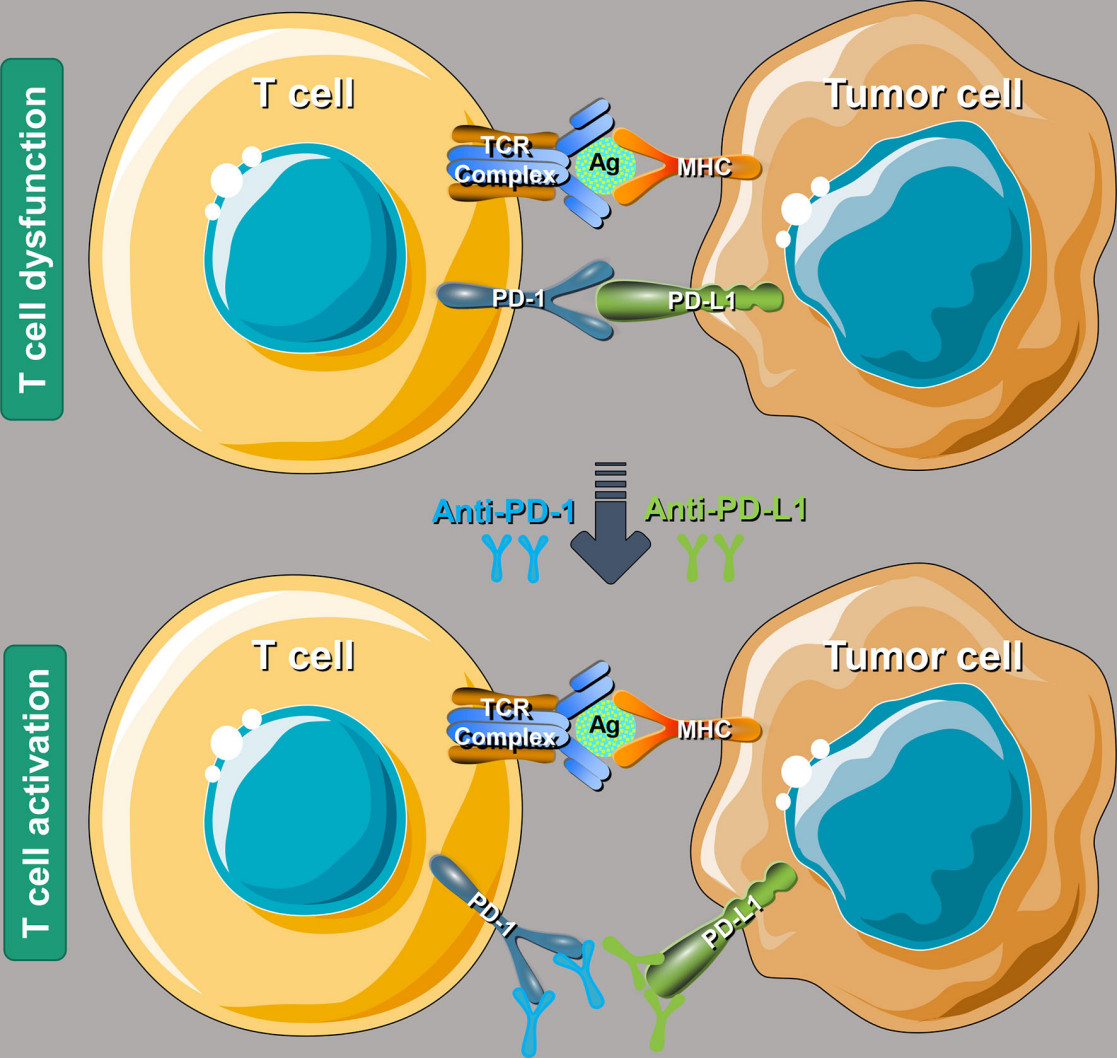
A schematic diagram of the molecular mechanism using PD-1/PD-L1 ICIs to rescue T cell functions. PD-1, programmed cell death-1; PD-L1, programmed death-ligand 1; ICIs, immune checkpoint inhibitors; TCR, T cell receptor; Ag, antigen; MHC, major histocompatibility complex.

After PD-1 binds to PD-L1, it will lead to the phosphorylation of the immunoreceptor tyrosine inhibitory motif (ITIM) and immunoreceptor tyrosine switching motif (TISM) of PD-1, and then inhibit T cell activation by recruitment of protein tyrosine phosphatase Src homology 1 domain tyrosine phosphatases (SHP-1) and SHP-2, or by up-regulating the expression of ATF-like alkaline leucine zipper transcription factor (BATF) (34). PD-1/PD-L1 immune checkpoint inhibitors (ICIs), by binding to PD-1 or PD-L1, block the binding of PD-L1 on the surface of tumor cells and PD-1 on the surface of immune cells, as shown in **Figure 1**, to relieve the inhibitory effect of tumor cells on the immune system and restore killing effect of the immune system on tumor cells (35, 36).

## Drug Resistance-Related Mechanisms of Anti-PD-1/PD-L1 Immunotherapy

### Attenuate Recognition of Tumor Antigens by Immune Cells

The frequency of gene mutations in tumor cells is high, which can cause abnormal expression of related molecules for processing and presenting tumor antigens, such as the downregulation of major histocompatibility complex (MHC)-I molecule expression. Abnormal expression of these molecules will cause immune cells to fail to recognize tumor cells usually, thereby mediating the immune tolerance of tumor-associated antigens, resulting in immune cells not being able to effectively identify and kill tumor cells (37, 38).

### Inhibit Activation of Immune Cells

Tumor cells can release various immunosuppressive factors, such as adenylate, indoleamine 2,3-dioxygenase 1 (IDO), prostaglandin E2 (PEG2), interleukin-10 (IL-10), and transforming growth factor-β (TGF-β), to inhibit the activation of immune cells (39). At the same time, tumor cells can also promote the expression of immunosuppressive molecules, such as PD-1, PD-L1, PD-L2, CTLA-4, and further inhibit the activation of immune cells (40).

### Promote Production of Suppressive Immune Cells and Molecules

Tumor cells can induce the production of immunosuppressive cells, such as T regulatory cells, natural killer T (NKT) cells, and bone marrow-derived immunosuppressive cells (41, 42). Immune cells can play an immunosuppressive role by contacting other cells and releasing corresponding inhibitory immunoregulatory factors. By up-regulating molecules related to the signal axis, such as PD-L1/PD-1, Fas ligand (FASL)/FAS, inhibitory regulatory factors can mediate the depletion of T cells (37).

### Affect Tumor Stromal Cells and Change TME

In tumor microenvironment (TME), tumor cells can promote the proliferation and aggregation of fibroblasts, macrophages, bone marrow-derived immunosuppressive cells, T regulatory cells, and NKT cells, and change tumors by contacting each other or secreting cytokines to inhibit the normal function of the immune system in killing tumor cells. TME may weaken the ability of the immune system to recognize and kill tumor cells (42, 43).

## Immunotherapy Efficacy Prediction Related Biomarkers

**Figure 2:**
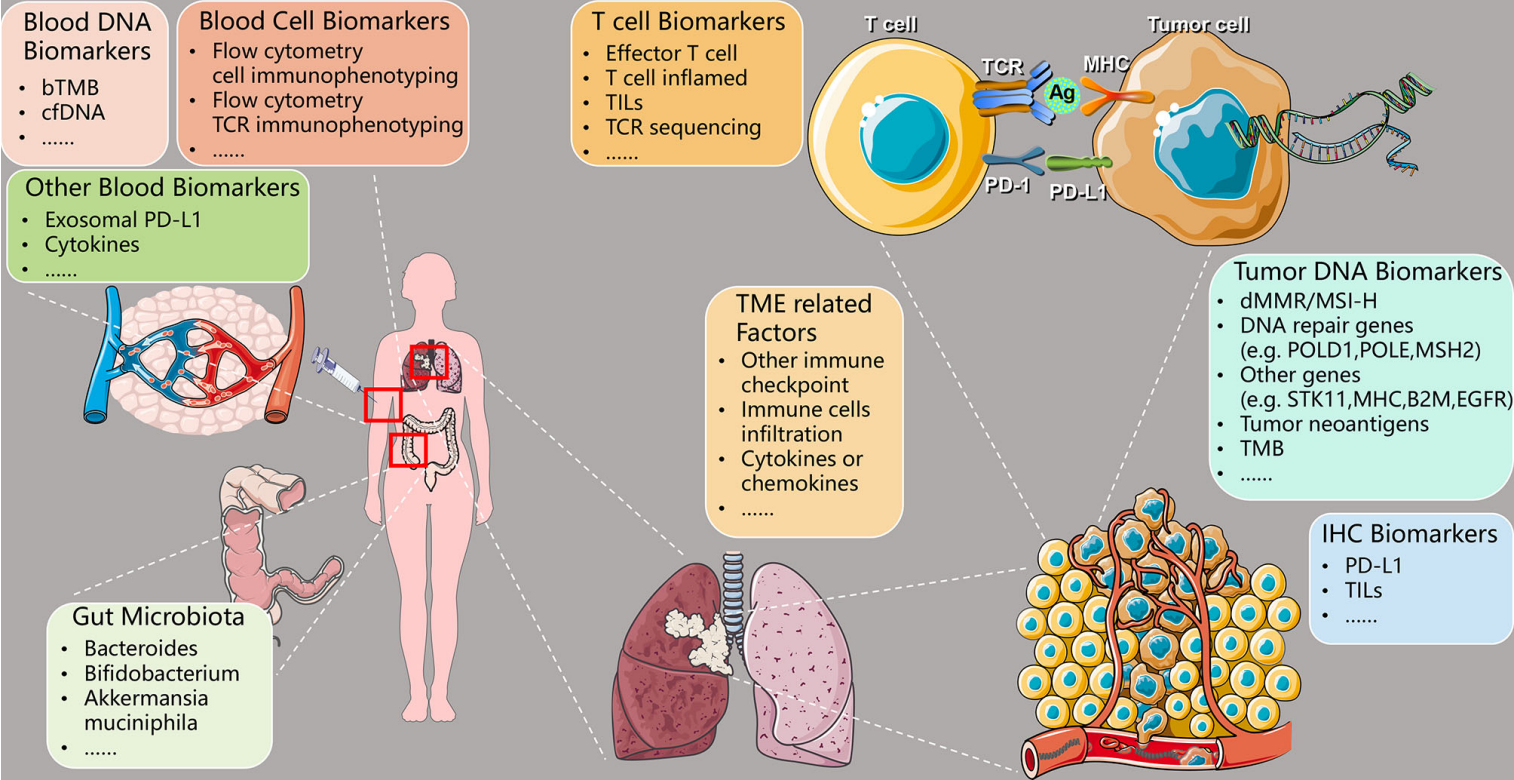
Predictive biomarkers of therapeutic efficacy using PD-1/PD-L1 ICIs in NSCLC. PD-1, programmed cell death-1; PD-L1, programmed death-ligand 1; ICIs, immune checkpoint inhibitors; NSCLC, non-small cell lung cancer; TCR, T cell receptor; Ag, antigen; MHC, major histocompatibility complex; bTMB, blood-based tumor mutational burden; cfDNA, circulating-free DNA; TILs, tumor-infiltrating lymphocytes; TME, tumor microenvironment; dMMR, deficient mismatch repair; MSI-H, microsatellite instability-high; *POLD1*, DNA polymerase delta 1, catalytic subunit; *POLE*, DNA polymerase epsilon, catalytic subunit; *MSH2*, mutS homolog 2; *STK11*, serine/threonine kinase 11; *B2M*, beta-2-microglobulin; *EGFR*, epidermal growth factor receptor; TMB, tumor mutational burden; IHC, immunohistochemistry.

Different NSCLC patients acquire different efficacy of anti-PD-1/PD-L1 immunotherapy due to individual differences. It is essential to find suitable biomarkers for predicting efficacy and screen out patients ideal for immunotherapy. At present, the commonly used clinical and immunological efficacy prediction biomarkers mainly include PD-L1 expression, TMB, microsatellite instability-high (MSI-H)/deficient mismatch repair (dMMR), etc. However, each has its corresponding shortcomings, so a combination of several biomarkers is usually used to improve the efficacy of predicting. This article focuses on the following predictive biomarkers related to anti-PD-1/PD-L1 immunotherapy (as shown in **Table 1** and **Figure 2**): PD-L1, TMB, MSI-H/dMMR, tumor DNA-related biomarkers, peripheral blood-related biomarkers, intestinal flora-related biomarkers, TME-related biomarkers, T cell-related biomarkers, and immunohistochemistry (IHC) related biomarkers.

**Table 1:** Summary of predictive biomarkers using PD-1/PD-L1 ICIs in NSCLC.

Category	Sub-Category	Biomarker	Example
Tumor	DNA Biomarkers	dMMR/MSI-H	**-**
		TMB	**-**
		DNA repair genes	*POLD1*, *POLE*, *MSH2*
		Other genes	*STK11*, *MHC*, *B2M*, *EGFR*
	Protein Biomarkers	PD-L1	**-**
		Tumor neoantigens	**-**
		Other immune checkpoints	CTLA-4, LAG3, TIM3
	TME related Factors	Immune cells infiltration	CD4+ T cells, CD8+ T cells
		Cytokines or chemokines	TGF, TNF, interleukin
		Stromal composition	Cancer-associated fibroblast
	T cell Biomarkers	Effector T cell	CD4+ T cells, CD8+ T cells
		T cell inflamed GEP	*CCL5*, *CXCL13*
		TILs	CD8+ T cells, NK cells
		TCR sequencing	CDR3
Blood	DNA Biomarkers	bTMB	**-**
		cfDNA	SNV, fragment, CNV
	Cell Biomarkers	Flow cytometry cell immunophenotyping	CD4+ T cells, CD8+ T cells
		Flow cytometry TCR immunophenotyping	CDR3
	Other Blood Biomarkers	Exosomal PD-L1	**-**
		Cytokines	TGF, TNF, interleukin
Gut Microbiota		Bacteroides	**-**
		Bifidobacterium	**-**
		Akkermansia muciniphila	**-**

PD-1, programmed cell death-1; PD-L1, programmed death-ligand 1; ICIs, immune checkpoint inhibitors; NSCLC, non-small cell lung cancer; dMMR, deficient mismatch repair; MSI-H, microsatellite instability-high; TMB, tumor mutational burden; POLD1, DNA polymerase delta 1, catalytic subunit; POLE, DNA polymerase epsilon, catalytic subunit; MSH2, mutS homolog 2; STK11, serine/threonine kinase 11; MHC, major histocompatibility complex; B2M, beta-2-microglobulin; EGFR, epidermal growth factor receptor; CTLA-4, cytotoxic T lymphocyte-associated molecule-4; LAG3, lymphocyte-activation gene 3; TIM3, T cell immunoglobulin and mucin domain-containing protein 3; TME, tumor microenvironment; CD, cluster of differentiation; TGF, transforming growth factor; TNF, tumor necrosis factor; GEP, gene expression profiling; CCL5, C-C motif chemokine ligand 5; CXCL13, C-X-C motif chemokine ligand 13; TILs, tumor-infiltrating lymphocytes; NK cell, natural killer cell; TCR, T cell receptor; CDR3, complementarity determining region 3; bTMB, blood tumor mutational burden; cfDNA, circulating-free DNA; SNV, single nucleotide variant; CNV, copy number variation.

### PD-L1 Expression Level

The expression level of PD-L1 in tumor tissues of patients is one of the most important biomarkers for whether patients choose ICIs therapy. At present, most studies have shown that NSCLC patients with high PD-L1 expression have a better therapeutic effect receiving anti-PD-1/PD-L1 immune treatment (44, 45). The commonly used indicator of PD-L1 expression level in NSCLC is TPS, TPS= (PD-L1 staining positive tumor cells)/(total live tumor cells) *100% (29). However, there is still controversy about the predictive efficacy of tumor PD-L1 expression. Studies have shown that the expression of PD-L1 in some NSCLC patients is low or even negative, but the effect of anti-PD-1/PD-L1 treatment is better (46). On the contrary, some patients have higher expression of PD-L1, and the therapeutic effect of anti-PD-1/PD-L1 therapy is poor.

### TMB

TMB has an excellent predictive value for the efficacy of immunotherapy. Studies have shown that TMB is an independent prognostic factor related to the effectiveness of immunotherapy and is not affected by the expression level of other indicators (such as PD-L1) (14). At present, many studies have shown patients with high TMB in tumors have a better therapeutic effect on anti-PD-1/PD-L1 immunotherapy (47, 48), and the same is true for NSCLC patients (49). Detection of TMB in the blood (bTMB) is also a new TMB detection method. Studies have shown that bTMB is related to the immune therapeutic efficacy of NSCLC (50), but more studies are still needed to verify it. Although both the expression level of PD-L1 and TMB are related to the efficacy of patients receiving immunotherapy, studies have shown that there is no exact correlation between PD-L1 expression and TMB (51).

### MSI-H/dMMR

Current studies have shown that MSI-H/dMMR can predict the efficacy of immunotherapy for gastric cancer and colon cancer, but the incidence of MSI-H/dMMR in lung cancer is low (52). Therefore, further research needs whether MSI-H/dMMR can be used as a predictive biomarker for NSCLC immunotherapy. At present, the standard measure commonly used to judge MSI-H is the Bethesda method (53). Research by Vanderwalde et al. showed that patients with MSI-H have a higher probability of having high TMB, but not vice versa (54).

### Tumor DNA-Related Biomarkers

In addition to classic prediction biomarkers of immune efficacy, such as TMB and MSI-H/dMMR described above, studies have shown that the existence of specific driver gene mutations is related to the effectiveness of immunotherapy. For example, the typically favorable genes in NSCLC are *TP53* (tumor protein p53), *KRAS* (KRAS proto-oncogene, GTPase), etc. Common negative genes include *EGFR* (epidermal growth factor receptor), *MET* (MET proto-oncogene, receptor tyrosine kinase), *ALK* (ALK receptor tyrosine kinase), etc. Patients with favorable gene mutations will have better therapeutic effects when receiving immunotherapy, while patients with negative gene mutations will have poorer efficacy. But a larger cohort is still needed for verification (19, 55). It has been reported that patients with driver gene mutations usually have lower TMB, while patients with high TMB usually have negative driver genes (56). Research by Garassino et al. showed that, regardless of *EGFR*/*ALK* mutation status, Durvalumab (anti-PD-L1 monoclonal antibody) has an excellent therapeutic effect for patients with greater or equal to 25% PD-L1 expression (57).

Tumor cells are prone to generate new mutations due to genome instability. The tumor-specific antigens produced by new mutations are called tumor neoantigens (58). Current studies have shown that tumor neoantigen is highly immunogenic and can activate CD4+ and CD8+ T cells to produce an immune response, which is expected to become a predictive biomarker in cancer immunotherapy (59).

### Peripheral Blood-Related Biomarkers

We divide peripheral blood-related immunotherapy efficacy biomarkers into three categories: blood DNA-related biomarkers, blood cell-related biomarkers, and other blood-related biomarkers.

Blood DNA-related biomarkers mainly include bTMB and circulating-free DNA (cfDNA). Wang Z et al. mainly used Next-Generation Sequencing technology and optimized algorithms to explore the relationship between bTMB and TMB in tumor tissue, indicating that bTMB can be used as an efficacy prediction biomarker of anti-PD-1/PD-L1 immunotherapy in NSCLC (60). The Brazos-Vázquez EM et al. team reviewed the application of liquid biopsy in immunotherapy of NSCLC patients, including circulating tumor cells (CTCs), cfDNA, and exosomes. It showed that liquid biopsy tools are expected to become promising predictive biomarkers for immunotherapy (61). Giroux Leprieur E et al. performed whole-exome sequencing on ctDNA to calculate bTMB and identify critical features, such as single nucleotide variants (SNVs), copy number variations (CNVs), to predict the efficacy of ICIs on advanced lung adenocarcinoma (62). Nabet BY et al. demonstrated that ctDNA and peripheral CD8+ T cell levels ahead of ICIs treatment are independently associated with durable clinical benefit for NSCLC patients receiving ICIs (63).

Blood cell-related biomarkers mainly include immune cell and T cell receptor (TCR) immunophenotyping by flow cytometry. The immune cells in peripheral blood can be classified through flow cytometry, and their number can be calculated (64). The type and number of immune cells (65), and TCR immunophenotyping (66), are both closely related to the efficacy of anti-PD-1/PD-L1 immunotherapy. Fumet JD et al. evaluated the role of CD8 under anti-PD-1 therapy and demonstrated that CD8 was a promising prognostic and predictive factor in NSCLC (67). The study result of Gettinger SN et al. showed CD3+ TILs related signal was associated with favorable response to ICIs therapy in NSCLC (68). The research of Anagnostou V et al. indicated that TCR clonal expansions within the tumor tissue or in circulating T cells were critical indicators to the therapeutic response of ICIs in NSCLC (69).

Other blood-related biomarkers mainly include exosomal PD-L1 and cytokines. Xie F et al. showed that exosomal PD-L1 plays a vital role in developing drug resistance in immunotherapy (70). Exosomal PD-L1 may potentially become a target for overcoming resistance to anti-PD-1/PD-L1 therapy (70). Current studies have shown that circulating exosomes play an essential role in developing tumors and the immune process of anti-tumor and have a good efficacy predictive value of patients receiving ICIs (71, 72). Interferon-gamma (IFN-γ), tumor necrosis factor (TNF), and other cytokines also play an essential role in the process of anti-PD-1/PD-L1 immunotherapy (73). All the above results indicate that peripheral blood-related biomarkers are promising indicators for predicting the therapeutic effect of ICIs.

### Biomarkers Related to Intestinal Flora

The predictive biomarkers related to intestinal flora of immunotherapy efficacy mainly include Bacteroides, Bifidobacterium, Akkermansia muciniphila. Vétizou M et al. showed that the anti-tumor effects of CTLA-4 blockers depend on the subclassification of Bacteroides species (74). The gut microbiome determines the different degrees to which ICIs can elicit the anti-cancer immune response (75). Current studies have shown that the type and quantity of intestinal microbes are closely related to the therapeutic effect of receiving ICIs.

### TME-Related Biomarkers

TME-related biomarkers mainly include other kinds of immune checkpoints, immune cells infiltration, and cytokines or chemokines. In addition to PD-1/PD-L1 ICs discussed explicitly in this article, common ICs include CTLA-4, LAG3, TIM3 (hepatitis A virus cellular receptor 2), and TIGIT (T cell immunoreceptor with Ig and ITIM domains), which also play essential roles in the process of anti-PD-1/PD-L1 immunotherapy (76, 77). The composition of immune cells in TME, such as CD8+ T cell, T regulatory cell, and the ingredient of cytokines and chemokines, are also closely related to the efficacy of anti-PD-1/PD-L1 immunotherapy (78, 79).

### T Cell-Related Biomarkers

T cell-related efficacy prediction biomarkers mainly include effector T cell, T cell inflamed gene expression profile (GEP), tumor-infiltrating lymphocytes (TILs), and TCR sequencing. The composition of effector T cells, such as CD4+ T cell, CD8+ T cell, T regulatory cell, T cell inflamed GEP, TILs composition, and TCR repertoire diversity, are closely related to the efficacy of anti-PD-1/PD-L1 immunotherapy (66, 78, 80). At present, most studies have shown that higher CD8+ T cell infiltration and lower T regulatory cell infiltration in tumor tissues indicate a better anti-PD-1/PD-L1 immunotherapy efficacy and better prognosis (81). The study by Han J et al. showed that TCR diversity and clonality in PD-1+ and CD8+ T cells of peripheral blood could be used as predictive biomarkers for the efficacy of anti-PD-1/PD-L1 immunotherapy (82).

## Conclusion

Immunotherapy is a vital tumor therapeutic method, which is widely used in the treatment of NSCLC. However, the immune system is one of the most complex systems in the human body, and the related mechanisms of immunotherapy are also very complex. Specific immunotherapy efficacy and drug resistance-related mechanisms still need a long time to explore. The most urgent problem is finding relatively stable predictive biomarkers for immunotherapy efficacy and screening out patients suitable for immunotherapy. It is believed that with the development of immunotherapy and biotechnology, immunotherapy will open up a new era of tumor therapy.

## Author Contributions

FB and SG designed this research. SG, HT, NS, RZ, and MZ collected articles. FB, PS, LL, YP, GB, and BZ wrote and polished the article. All of the authors reviewed the manuscript. All authors contributed to the article and approved the submitted version.

## Funding

This work was supported by the Institutional Fundamental Research Funds (2018PT32033) and the Ministry of Education Innovation Team Development Project (IRT-17R10).

## Conflict of Interest

The authors declare that the research was conducted in the absence of any commercial or financial relationships that could be construed as a potential conflict of interest.

## Publisher’s Note

All claims expressed in this article are solely those of the authors and do not necessarily represent those of their affiliated organizations, or those of the publisher, the editors and the reviewers. Any product that may be evaluated in this article, or claim that may be made by its manufacturer, is not guaranteed or endorsed by the publisher.
